# Early PCR-based DNA identification in a wartime mass-fatality event: archival reconstruction of the 1994 Duboki Jarak explosion

**DOI:** 10.3325/cmj.2026.67.175

**Published:** 2026-06

**Authors:** Dragan Primorac, Šimun Anđelinović, Ante Gugić, Ivan Mario Staničić, Marija Definis, Ivana Kružić, Ivan Jerković, Željana Bašić

**Affiliations:** 1 St. Catherine Specialty Hospital, Zabok/Zagreb, Croatia; 2 University of Pittsburgh School of Medicine, Pittsburgh, PA, USA; 3 Eberly College of Science, The Pennsylvania State University, State College, PA, USA; 4 School of Medicine, University of Split, Split, Croatia; 5 School of Medicine, Josip Juraj Strossmayer University of Osijek, Osijek, Croatia; 6 Henry C. Lee College of Criminal Justice and Forensic Sciences, University of New Haven, New Haven, CT, USA; 7 Sana Medical School, Coburg, Germany; 8 School of Medicine, University of Rijeka, Rijeka, Croatia; 9 Faculty of Dental Medicine and Health Osijek, Josip Juraj Strossmayer University of Osijek, Osijek, Croatia; 10 School of Medicine, University of Mostar, Mostar, Bosnia and Herzegovina; 11 National Forensic Sciences University, Gandhinagar, India; 12 University Hospital of Split, Split, Croatia; 13 Croatian Generals Association, Zagreb, Croatia; 14 Faculty of Forensic Sciences, University of Split, Split, Croatia; * These authors contributed equally and share first authorship.; ‡ These authors contributed equally and share senior authorship.

## Abstract

**Aim:**

To reconstruct the forensic identification process following the 1994 explosion at the Duboki Jarak military ammunition and explosive ordnance storage facility in Croatia and to evaluate the role of early PCR-based DNA typing in the identification of severely fragmented human remains under wartime conditions.

**Methods:**

A retrospective archival analysis was conducted using original forensic, judicial, and laboratory documentation generated after the explosion. We reviewed crime scene records, medico-legal documentation, forensic body diagrams, situational plans, judicial orders authorizing DNA testing, laboratory reports, and genotype summary tables. Archived laboratory records indicate that recovered tissue samples underwent PCR-based DNA typing using the GeneAmp PCR System 9600 with reverse hybridization assays targeting six loci (HLA-DQA1, LDLR, GYPA, HBGG, D7S8, and GC). The obtained profiles were compared with reference samples from the missing persons’ relatives.

**Results:**

Archival documentation recorded 59 biological specimens recovered at the scene. Of the 48 samples submitted for laboratory examination, 33 were subjected to PCR-based DNA typing, and 20 yielded interpretable DNA profiles. Among the recovered remains, multilocus comparison documented five distinct genetic profiles. Comparison with 16 reference samples from 16 relatives representing six families enabled kinship-based attribution of remains to five victims. No compatible genotype was identified in one family, and one specimen was excluded from attribution due to mixed PCR signals consistent with contamination.

**Conclusion:**

The Duboki Jarak case represents one of the earliest documented applications of PCR-based DNA typing for victim identification in a mass-fatality event during active wartime conditions. DNA-supported identification was operationally implemented in Croatia in 1994, several years before molecular genetics was formally integrated into international disaster victim identification protocols.

DNA identification was used for the first time in criminal cases in 1986, only two years after Sir Alec Jeffreys introduced DNA fingerprinting (1,2). Its value was quickly proven both as a means of exclusion and identification. The first forensic use of DNA in active policing was in the high-profile Pitchfork case (3).

In those early years, DNA was not yet used for mass-casualty identification. Interestingly, the same year that Jeffreys' method appeared (1984), the Interpol Disaster Victim Identification (DVI) Guide was first issued. However, as DNA identification was in its infancy, the methods at that time relied mainly on fingerprints, dental records, and anthropological comparison (4).

The first mass disaster in which DNA profiling was used was the Scandinavian Star ferry fire in 1990 (5), although the primary published accounts from the time do not reference DNA identification (6,7). Other early uses of DNA identification include the 1992 Airbus A320 crash on Mount Ste-Odile (8), the Waco disaster (9), and 1994 AMIA bombing in Buenos Aires (10). In the 1996 Spitsbergen aircraft disaster, DNA profiling became the primary identification method (11). Finally, in 1997, DNA analysis was formally integrated into international DVI protocols. This fundamentally changed identification practices by allowing structured kinship-based comparisons and active participation of relatives in the DVI process (12).

DNA profiling rapidly became essential for resolving complex mass-fatality events worldwide. This shift enabled structured kinship comparisons, reliable matching of highly fragmented or degraded remains, and greater involvement of relatives, leading to successful identifications in numerous high-profile incidents. Key examples include the Swissair Flight 111 crash in 1998 (13,14); the Taoyuan Airbus crash in 1998 (15); the Kaprun cable car fire in 2000 (16); the World Trade Center attacks in 2001 (17); the Bali bombings in 2002 (18); the Madrid train bombings in 2004 (19); the London 7/7 bombings in 2005 (20); the Oslo government quarter bombing and Utøya shootings in 2011 (21); and the Brussels Airport bombings in 2016 (22). These cases demonstrated DNA's transformative role in achieving accurate individualization under extreme fragmentation, commingling, and time pressures, a progress rooted in the methodological transformations of the 1990s.

Although widespread routine application of these methods became established internationally during the 2000s, Croatia had been facing comparable forensic challenges earlier, during the Homeland War (1991-1995), a period characterized by extensive destruction, large-scale human losses, and numerous mass graves (23-27). Emerging forensic technologies, therefore, were not implemented in stable institutional settings but under conditions of operational urgency, damaged infrastructure, and sustained humanitarian pressure to identify victims (25,27-30).

Within this fragile and fast-changing institutional environment, the explosion at the military ammunition and explosive ordnance storage facility in Duboki Jarak (Gliboki Jarek) near Sesvete, Zagreb, on April 7, 1994 was a particularly sudden and serious mass-fatality incident. The facility, previously captured from the Yugoslav People's Army in 1991 and now part of Croatia’s national defense infrastructure during an active phase of the Homeland War, stored large quantities of ammunition and explosives. The detonation occurred inside a secured military complex, causing multiple fatalities among personnel present at the site and generating complex forensic conditions due to high-pressure blast forces, intense thermal effects, and secondary fragmentation. These mechanisms resulted in severe disruption of anatomical integrity and extensive commingling of biological and non-biological material. Because of the strategic importance of such facilities and the wartime conditions, the incident demanded not only a forensic investigation but also a coordinated response at the military and state levels.

The Duboki Jarak explosion occurred at a time when polymerase chain reaction (PCR) technology had only recently been deployed in Croatia, and international DVI protocols had not yet been formalized. This context, wartime necessity, nascent forensic infrastructure, and severe taphonomic challenges make the case an important early example of operational forensic molecular genetics. The aim of this study is therefore to reconstruct for the first time the forensic response to the explosion, encompassing field recovery procedures and PCR-based DNA identification, and to critically evaluate this early implementation within the broader historical development of DVI practice.

## Materials and methods

### Study design

In this retrospective archival analysis, we reviewed the forensic documentation generated following the 1994 explosion at the military ammunition and explosive ordnance storage facility in Duboki Jarak. The victim identification procedure was reconstructed exclusively through primary written and graphic records, comprising the following: crime scene examination records and diagrams (April 16, 1994); medico-legal body diagrams; the Official Note on the Coordination Meeting and Initial Expert Inspection; the Order of the Military Investigating Judge Authorizing DNA Identification Analysis; the Summary Report of DNA Identification of Tissue Samples; DNA Typing Result Sheets (AMPLITYPE DQ-Alpha Test Strip Documentation); Official Correspondence to the Minister of Defense of the Republic of Croatia, Gojko Šušak; the Forensic Expert Report on DNA Identification of Human Remains from Duboki Jarak; and Official Communication from the Military Court in Zagreb to the Institute of Forensic Medicine, Zagreb.

### Reconstruction of procedural chronology

Procedural chronology was reconstructed through cross-comparison of dates, administrative markings, and sample identifiers, establishing formal linkage between scene investigation, sample registration, judicial authorization, and laboratory reporting.

### Scene documentation analysis

Scene documentation was evaluated through textual descriptions and graphical depictions of the explosion-affected sector. Recorded recovery locations of biological material were examined in relation to structural elements and marked on the situational plan.

### Medico-legal documentation review

Medico-legal documentation was examined to determine the scope of recorded postmortem and antemortem data. Postmortem forms documented biological and identifying parameters including sex, age, stature, body constitution, preservation status, traumas, healed injuries, scars, tattoos, dental status, and blood group. Antemortem information collected from families included physical descriptions, medical history, clothing at the time of disappearance, personal effects, and reference biological samples for comparative analysis.

### DNA documentation analysis

Archived DNA documentation was systematically reviewed to reconstruct the procedural workflow of the molecular identification performed following the Duboki Jarak explosion. The analysis was based exclusively on official archival records, including laboratory reports, genotype summary tables, and judicial authorization documents. No raw electrophoretic or primary genetic data were available.

Tissue samples recovered during the scene examination were subjected to laboratory processing that included mechanical homogenization of the biological material, DNA extraction and purification, PCR amplification, and subsequent allele detection by reverse hybridization. PCR amplification was performed using the GeneAmp PCR System 9600 thermal cycler (Perkin Elmer, Shelton, CT, USA). Genotyping was carried out using DQA1 and Polymarker reverse hybridization kits (Perkin Elmer) targeting six loci commonly used in early forensic PCR systems: HLA-DQA1, LDLR, GYPA, HBGG, D7S8, and GC. DNA analysis was performed at the Laboratory for Clinical and Forensic Genetics, Department of Clinical Pathology, University Hospital of Split.

The documentation indicated that, due to advanced degradation of many samples, attributed to postmortem decomposition, high temperatures, and blast-related tissue disruption, additional purification steps were frequently required prior to amplification. Following hybridization-based allele detection, the results were compared with DNA profiles obtained from biological relatives of the missing individuals.

Statistical evaluation of genotype compatibility was based on calculations under the Hardy-Weinberg equilibrium and related population genetic principles. In selected cases, supplementary identification approaches were applied, including classical morphological recognition methods, ABO blood group determination to assess human specificity, and gas chromatography analysis of alcohol concentration in tissue samples.

The archived records were further examined to evaluate sample traceability, the number of amplification attempts, consistency of genotype reporting, and the documented linkage between laboratory findings and family reference comparisons used for identification.

### Data anonymization

Personal identifiers were anonymized throughout the article. Highly specific descriptions of distinctive personal characteristics that could allow recognition of individual victims were intentionally omitted to preserve the dignity of the deceased and the privacy of their families.

## Results

### Forensic response and scene findings

**Figure 1:**
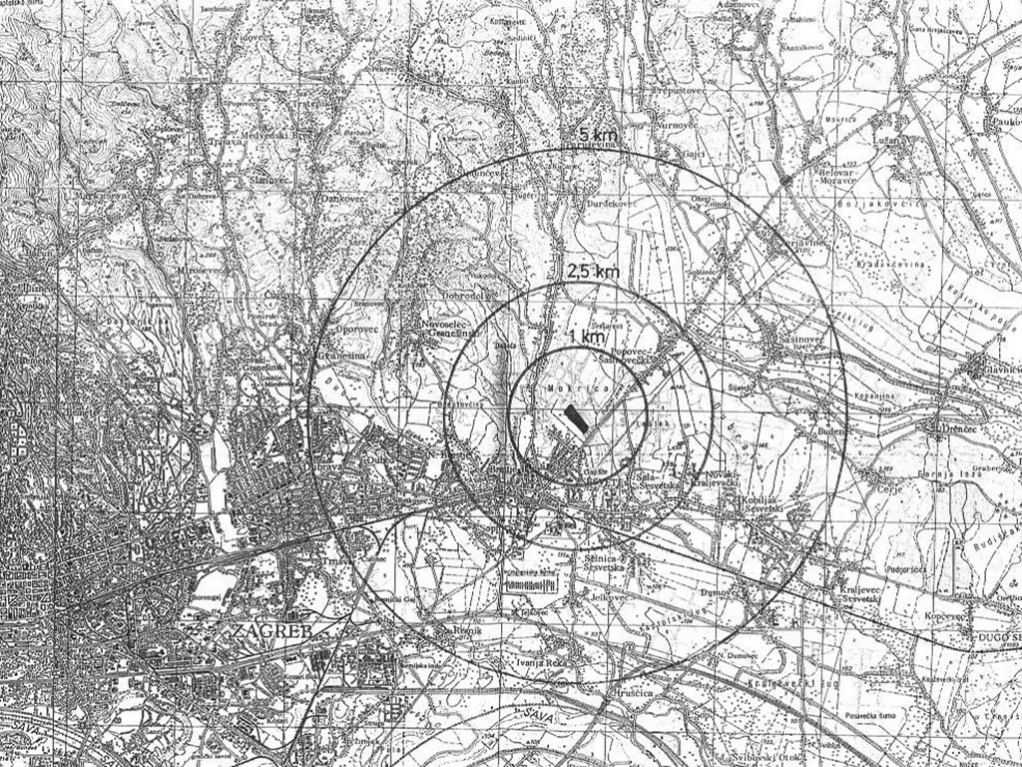
Geographic location of the Duboki Jarak military storage facility near Sesvete (Zagreb, Croatia).

The explosion occurred on April 7, 1994, at the military explosives’ storage facility in Duboki Jarak (Figure 1). The primary response was to secure the scene, evacuate residents in the surrounding areas, monitor and evaluate safety, and rule out the possibility of secondary explosions, as well as the presence of chemical warfare agents. On April 16, 1994, a coordination meeting and restricted on-site inspection were conducted under the authority of the investigating judge. Participants included fire and explosion experts (Center for Criminalistic Examinations), a ballistics expert, a biological trace expert, criminal technicians of the Zagreb Police Directorate, a representative of the Faculty of Criminalistics, and experts from the Institutes of Forensic Medicine in Zagreb and Split. Croatian Army specialists were tasked with clearing the terrain and securing the site.

**Figure 2:**
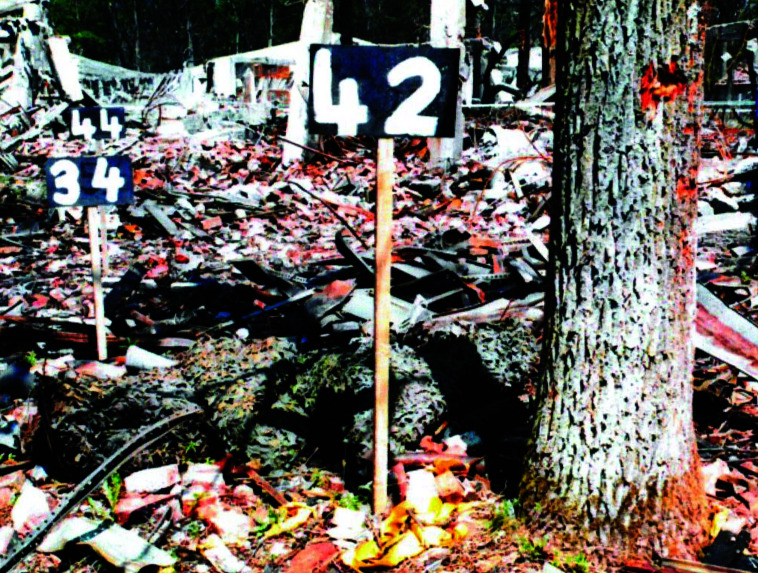
General view of the Duboki Jarak site following the explosion, illustrating the overall extent of destruction.

The site was inspected exclusively along previously cleaned routes, as all the object facilities were razed to the ground (Figure 2). The inspection mostly focused on the location previously known to be occupied by army personnel, which had suffered the most extensive damage and was assumed to be the initial point of the explosion. The entire facility was subdivided into seven sectors, and a crime-scene investigation plan was developed. In the next phase, the whole scene was thoroughly examined by military investigating officers and by an interdisciplinary team of forensic experts, depending on the priorities and evidence types. Before the collection of biological evidence, forensic experts provided additional training to military personnel for handling such specimens. On-site points were established for initial triage and processing of evidence.

**Figure 3:**
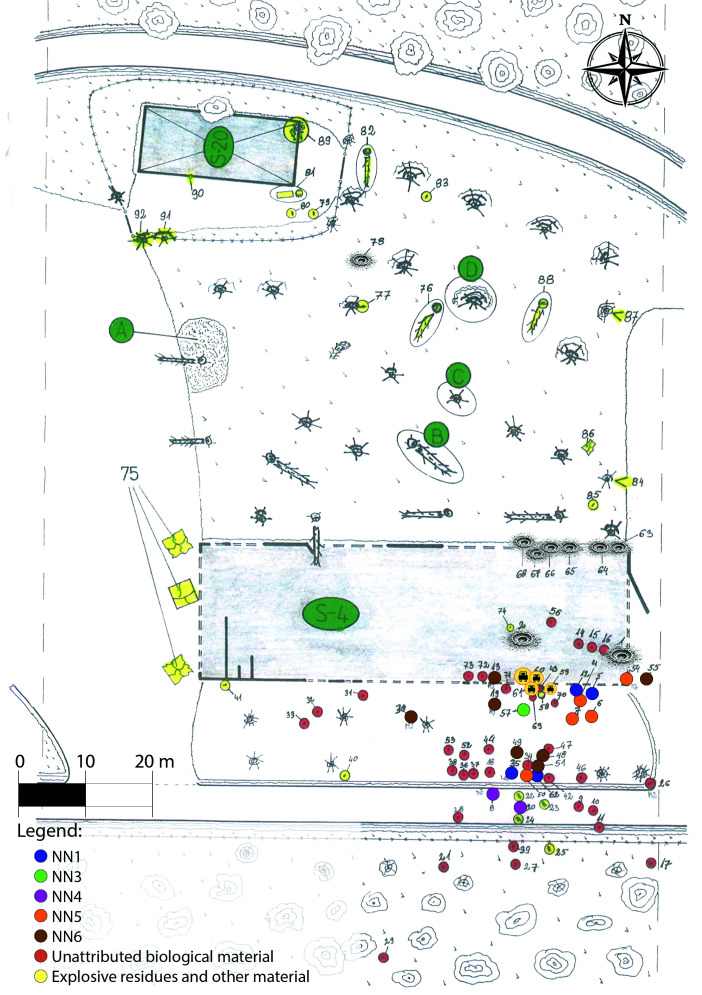
Sector 3 of the Duboki Jarak ammunition storage facility showing the spatial distribution of recovered biological material and other evidence.

The highest priority was assigned to Sector 3, corresponding to the center of the explosion and the origin of the victims (Figure 3). It was completely destroyed, with most of the items of explosive ordnance and biological evidence found in the southeast corner of warehouse 4 (S-4). The area north of the S-4 showed traces of vast devastation of trees, with burned and exploded components of explosive ordnance scattered across the terrain. Warehouse 20 (S-20), situated in the northwest part of sector 3, was also significantly damaged, and several craters were observed. The initial explosion, occurring in S-4 was caused by forklift handling . Specifically, based on the observed lowered position of the forks, it was concluded that the forks had mechanically activated detonator-equipped explosive ordnance. The lowered position of the forks indicated that the operator was depositing or moving explosive ordnance within the storage area, which allowed mechanical contact with ordnance that still had detonators attached. During the extended examination of the wider scene, dispersed human remains were recovered and subsequently associated with the forklift operator. The operator was likely propelled from the forklift by the blast pressure and expelled through a window opening, with the body later recovered in a nearby stream. It was the only victim recovered in a relatively preserved state, having been ejected outside the burning structure and thus not exposed to the intense thermal destruction affecting the other victims. Given the large quantities of stored munitions within the facility, the initial detonation likely triggered subsequent secondary explosions. Under such storage conditions, moving detonator-equipped explosive ordnance using an industrial forklift represented a highly hazardous practice. Most of the fragmented biological evidence was found in the southern part of the building, mostly focused on the initial explosion radius, while less evidence was scattered in a wider area.

### Biological samples collected from the scene

Biological material was collected during two formal scene examinations conducted on April 16 and April 28, 1994 in warehouse S-4. Thirteen tissue samples were recovered during the first examination (DJ-1/94 to DJ-13/94), and an additional 5 samples (DJ-14/94 to DJ-18/94) during the second examination. Further fragments were collected during continued terrain processing and assigned consecutive DJ numbers, resulting in 59 documented specimens.

Most recovered specimens consisted of dismembered body parts and isolated soft tissue and bone fragments dispersed around and within warehouse S-4. Macroscopic examination demonstrated extensive thermal damage, carbonization, blast-related fragmentation, and varying degrees of postmortem decomposition. Anatomical elements identified among the fragments included portions of long bones, vertebrae, pelvic bones, ribs, distal extremities, and variable amounts of soft tissue. In several specimens, preserved muscle, bone, or hair allowed sampling for forensic analysis, whereas in others, severe burning and advanced degradation rendered the material unsuitable for further examination.

In addition to the fragmented biological material summarized in Table 1, one largely preserved body (DJ-13/94) was recovered from a drainage canal adjacent to the road. The body was found in prone position, partially submerged in water, with the dorsal trunk protruding above the surface. Advanced traumatic disruption was present, including near-complete decapitation and absence of the distal portions of both lower legs. The body was of robust osteomuscular constitution and was clothed in trousers and an undershirt heavily contaminated with mud. Blood, muscle, and bone samples were collected for laboratory analysis, and a fingerprint of the right index finger was obtained for comparison. The body was subsequently transferred to the Institute of Forensic Medicine in Zagreb for autopsy and classical identification procedures.

**Table 1:** Biological material recovered at Duboki Jarak (DJ-1/94–DJ-59/94) and submitted for laboratory examination

DJ No.	Police No.	Location	Size (cm)	Anatomical description	Macroscopic condition	DNA sampled	Serology sampled	DNA suitable	Remarks
DJ-1/94	6	4.2 m S of SW edge S4	—	Charred muscle +7 vertebrae (lumbo-sacral)	Heavily carbonized, soil contamination	Muscle + vertebra	Muscle	Yes	—
DJ-2/94	4	4 m SW of S4	60 × 40	Bilateral thighs + pelvis + penis remnants	Extensive burns	Femur + hair	Muscle	Yes	Male, robust build
DJ-3/94	7	6 m S of SW edge S4	20 × 13	Skin/subcutis + muscle	Severely charred	Muscle	Muscle	Yes	—
DJ-4/94	5	Road edge SW of S4	35 × 18	Right foot including hallux	Carbonized	Tibial bone + muscle + hair	Muscle	Yes	—
DJ-5/94	12	0.5 m S of SW edge S4	32 × 14	Charred muscle + bone fragments	Carbonized	Long bone	Muscle	Yes	—
DJ-6/94	13	Inside S4 (W of forklift)	—	Pelvic fragments + femoral head	Charred, metal fragment in bone	Bone + muscle	Muscle	Yes	Embedded metal fragment
DJ-7/94	16	Near explosion epicenter	7 × 5	Charred muscle + bone	Severe carbonization	—	—	No	Unsuitable
DJ-8/94	14	W of No.16	—	Scapular fragment	Severely burned	—	—	No	Unsuitable
DJ-9/94	16	Inside S4	18 × 14	Skin/subcutis	Heavily carbonized	—	—	No	Unsuitable
DJ-10/94	10	Outside S4 near road	8 × 6	Vertebral fragments	Burned	Vertebra	—	Yes	—
DJ-11/94	11	Roadside near ditch	12 × 6	Muscle	Burned, maggot activity	Muscle	Muscle	Yes	—
DJ-12/94	9	Road under S4	—	Small bone fragment (prob. vertebra)	Severely burned	—	—	No	Unsuitable
DJ-13/94	8	Drainage ditch	—	Body (male)	Advanced trauma, partial decapitation	Blood + muscle + bone	Muscle + blood	Yes	Fingerprint taken; autopsy Zagreb
DJ-14/94	17	S side S4	26 × 25	Left pelvis + proximal femur	Charred soft tissue	Bone	—	Yes	—
DJ-15/94	18	Ditch in front of S4	12 × 10	Charred tissue + bone	Burned	Bone	—	Yes	—
DJ-16/94	19	S of S4 near forklift	18 × 15	Muscle	Heavily carbonized	Muscle	Muscle	Yes	—
DJ-17/94	20	Road 1.5 m from ditch	12 × 8	Muscle + joint surface bone + skin	Carbonized	Bone + muscle	—	Yes	—
DJ-18/94	21	Outside fence S of S4	—	Burned skin	Carbonized	—	—	No	Unsuitable
DJ-19/94	B39	Sector B39 S of S4	5 × 3.5	2 vertebrae + bone	Carbonized	Bone	—	Yes	—
DJ-21/94	26	Drainage canal S of S4	60 × 45	Right arm + chest	Decomposed, soot	Bone + muscle	—	Yes	—
DJ-22/94	27	Behind fence	11 × 10	Knee joint (femur + tibia)	Carbonized	Femur	—	Yes	—
DJ-24/94	29	Forest behind fence	21 × 10	Dried charred skin	Carbonized	—	—	No	Unsuitable
DJ-25/94	30	W of forklift	—	Right arm	Burned, clothing present	Bone + muscle	—	Yes	Ring impression
DJ-32/94	35	1 m from canal	25 × 10	Proximal tibia/fibula	Burned soft tissue	Bone + muscle	Muscle	Yes	—
DJ-35/94	38	2.3 m NE road	10 × 6 × 3.5	Bone + soft tissue	Burned/decomposed	Bone	—	Attempted	—
DJ-37/94	41	—	40 × 27	Skin + subcutis	Decomposed	—	—	No	Unsuitable
DJ-38/94	42	—	11 & 14	Finger + metacarpals	Burned	Bone + muscle	—	Yes	—
DJ-39/94	43	0.4 m S SW S4	—	Vertebrae	Burned	Bone	—	Yes	—
DJ-40/94	44	—	18 × 3	Soft tissue	Carbonized	—	—	No	Unsuitable
DJ-41/94	45	Crater No.2	5 × 3	Stone	Inorganic	—	—	No	Not biological
DJ-42/94	46	1 m NE road	10 × 4.5	Fatty subcutis	Carbonized	—	—	No	Unsuitable
DJ-43/94	47	10 m S of SW S4	10 × 7 × 3.5	Soft tissue	Carbonized	—	—	No	Unsuitable
DJ-44/94	48	11 m S of SW S4	—	Fragments of long bones and knee joint	Burned	Bone	—	Yes	—
DJ-45/94	49	—	—	Part of right foot with skin	—	Bone	—	Yes	—
DJ-46/94	50	2 m NE of road	12 × 7	Part of soft tissue with bone (scapula)	Burned	Bone	—	Yes	—
DJ-47/94	51	4 m NE of road	—	Soft tissue with bone (ankle)	Burned	Bone	—	Yes	—
DJ-48/94	52	—	9 cm, 8cm	Soft tissue, bone fragments	Carbonized	—	—	No	Unsuitable
DJ-49/94	53	On field, 6 m NE from road	8 × 3	Soft tissue	Carbonized	—	—	No	Unsuitable
DJ-50/94	54	SE edge of S4	3.5 × 2.7	Maxilla with four teeth	—	Tooth	—	Yes	—
DJ-51/94	55	Gravel plateau SE entrance	7 × 2	Long bone	Burned	Bone	—	Yes	—
DJ-52/94	56	10 m SW concrete edge	17 × 10	Soft tissue + long bone	Carbonized, maggots	—	—	No	Unsuitable
DJ-53/94	57	4 m S of S4	26 & 7	Soft tissue + long bone	Burned	Bone	—	Yes	—
DJ-54/94	62	0.7 m from road	17 & 7	Long tubular bone	Burned	Bone	—	Yes	—
DJ-55/94	69	Crater 2 m S SW S4	—	Pelvis + proximal femur	Burned	Bone	—	Yes	—
DJ-56/94	70	4 m S SW S4	—	Ribs + soft tissue	Burned	Rib bone	—	Yes	—
DJ-57/94	71	0.6 m S SW S4	17 × 9	Burned skin	Carbonized	—	—	No	Unsuitable
DJ-58/94	72	2 m NE SW S4	50 × 25	Chest + abdominal wall + ribs	Decomposed	Bone	—	Yes	—
DJ-59/94	73	2 m NE SW S4	9 × 3.5	Scar tissue + hair	Decomposed	—	—	No	Unsuitable

In one specimen (DJ-6/94), a metallic fragment was identified embedded within the pelvic bone, consistent with high-energy blast-related projectile impact. This finding represents direct evidence of explosive fragmentation injury.

Certain fragments demonstrated potential identifying features beyond routine anatomical attribution. In DJ-25/94 (right upper limb), a circular impression consistent with prior ring wear was observed on the third finger. Clothing remnants were present on several specimens, including a camouflage-pattern textile and blue long-sleeved fabric. In DJ-59/94, light brown hair measuring up to 3 cm in length was documented; however, advanced postmortem alteration precluded further analysis.

Several documented specimen numbers corresponded to material that was not received or to empty evidence bags, indicating either retention at another forensic institution or loss prior to laboratory submission. One documented item (DJ-41/94) was inorganic material (stone) and was excluded from biological analysis. A separate unlabeled fragment of carbonized muscle tissue (30 × 8 cm) was documented but was unsuitable for laboratory examination.

### Antemortem data and reference samples

As the victims were military personnel, antemortem information was collected by military police investigating officers. Available personal and service records were obtained, and additional information was gathered through interviews with the victims’ family members, military superiors, and colleagues. The collected data included demographic characteristics, physical descriptions, dental information, medical history, and information on clothing and personal belongings at the time of disappearance. Several victims presented potentially distinctive identifying features, including tattoos, scars, dental anomalies, and documented blood group information. The available antemortem profiles for six victims are summarized in Table 2.

**Table 2:** Summary of available antemortem information for the six victims

Variable	NN1	NN2	NN3	NN4	NN5	NN 6
**Age (years)**	39	46	39	27	35	20
**Height (cm)**	172	185	185	173	184	180
**Weight (kg)**	82	85	105	95	80	80
**Mother alive**	Yes	Yes	No	Yes	Yes	Yes
**Father alive**	No	Yes	Yes	Yes	Yes	Yes
**Children**	Yes (3)	Yes (1)	Yes (2)	Yes (1)	Yes (2)	No
**Hair**	Chestnut, short	Blond, short	Brown, short, straight	Chestnut, short, straight	Brown, short, straight	Dark brown, short, straight
**Dentition**	Missing upper anterior teeth (2, 3)	Missing teeth (unspecified)	N/A	Right upper tooth without crown	Poor overall dentition: posterior teeth affected	Anterior teeth intact; posterior caries present
**General physical characteristics**	Prominent zygomatic regions; multiple scars over body; scar above right upper lip	Partial frontal alopecia	Broad brown moustache	No specific distinguishing traits	No specific distinguishing traits	No specific distinguishing traits
**Healed fractures / injuries**	Burn scar on right arm and shoulder	None reported	Healed fracture of right ankle	Healed left knee meniscal rupture	None reported	None reported
**Scars**	Multiple cutaneous scars	None reported	Two forehead scars ( ~ 2 cm each)	None reported	2 cm scar above left zygomatic bone; medial scar on left knee (sutured wound)	None reported
**Tattoos**	Inscription above right wrist	None reported	Inscription on inner forearm	None reported	None reported	None reported
**Clothing at disappearance**	Black shoes; camouflage shirt, trousers, vest; military socks; black short-sleeved undershirt	Lace-up military boots; green socks and trousers; camouflage jacket	Camouflage trousers and shirt; gray high-collar vest with zipper; black military boots; white socks	Military boots; camouflage trousers; gray shirt and jacket; white undershirt	Camouflage trousers and jacket; gray vest; brown lace-up shoes; white undershirt	Shoes; camouflage uniform
**Personal effects**	Necklace; wristwatch (left wrist)	Thick gold chain; wide woven bracelet	Gold chain with small crucifix ( ~ 1 × 2 cm)	Wristwatch with damaged leather strap (in pocket)	White gold ring with engraved date and name; gray wristwatch	Wristwatch (in pocket or worn)
**Blood group**	AB Rh+	Not recorded	A Rh+	O Rh+	B Rh+	O Rh (?)

Reference samples were collected from members of six families, comprising a total of 16 individuals. The samples were obtained from parents, spouses, and children of the missing persons and were used for comparative genetic analysis (Table 3).

**Table 3:** Summary of reference samples by family

Family	Sampled family members	Number of samples
NN1	mother, wife, son, daughter	4
NN2	son, wife	2
NN3	father, wife, two daughters	4
NN4	mother, father	2
NN5	mother, father	2
NN6	mother, father	2
**Total**	—	**16**

### DNA analysis

Of 59 collected tissue samples, 11 were not submitted for laboratory analysis, and the authorized Military Court judge was informed accordingly. Of the 48 samples received for examination, 15 were excluded prior to molecular analysis due to advanced decomposition and extensive carbonization.

The remaining 33 samples were mechanically homogenized, and DNA was isolated and amplified by PCR (GeneAmp PCR System 9600, Perkin Elmer). Allele detection was performed by reverse hybridization using DQA1 and Polymarker kits (Perkin Elmer). Because of decomposition, thermal exposure, and blast-related damage, DNA extracts were frequently degraded and required additional purification prior to amplification.

Of the 33 samples, 13 did not yield amplifiable DNA despite repeated testing. Successful DNA profiling was achieved in 20 samples (61% of processed samples; 42% of all received samples). All successfully analyzed specimens were confirmed to be of human origin.

Table 4 presents the multilocus DNA profiles obtained from the 20 successfully analyzed samples. Each column represents a genetic locus (HLA-DQA1, LDLR, GYPA, HBGG, D7S8, and GC), and each row represents a unique genotype. At every locus, two alleles are recorded, reflecting the diploid nature of nuclear DNA. The DQA1 values (eg, 1.2; 4) denote specific sequence-defined variants of the HLA class II gene, while the letter-coded results at the remaining loci indicate alternative allelic forms detected by locus-specific probes. The combined allele pattern across all six loci constitutes an individual genetic profile.

**Table 4:** Genetic loci profiles of five identified genotypes

Genotype	DQA1	LDLR	GYPA	HBGG	D7S8	GC
1	1.2; 4	AB	AB	AB	AB	AC
2	1.3; 2	AB	BB	AB	AB	AC
3	1.2; 4	AB	AB	AB	AA	AC
4	1.2; 4	AB	AA	AB	AB	BC
5	1.1; 4	AA	AB	AB	AD	AC

Comparison of the composite multilocus profiles demonstrated five distinct genotypes. Five samples (DJ 1, 2, 3, 46, and 50) shared one identical genotype; three samples (DJ 13, 17, and 21) shared a second genotype; six samples (DJ 6, 25, 44, 45, 47, and 51) shared a third genotype; five samples (DJ 4, 5, 32, 54, and 55) shared a fourth genotype; and one sample (DJ 53) exhibited a unique fifth genotype. These findings confirmed the presence of five genetically distinct individuals.

### Linking DNA genotypes of families with genotypes of samples

**Figure 4:**
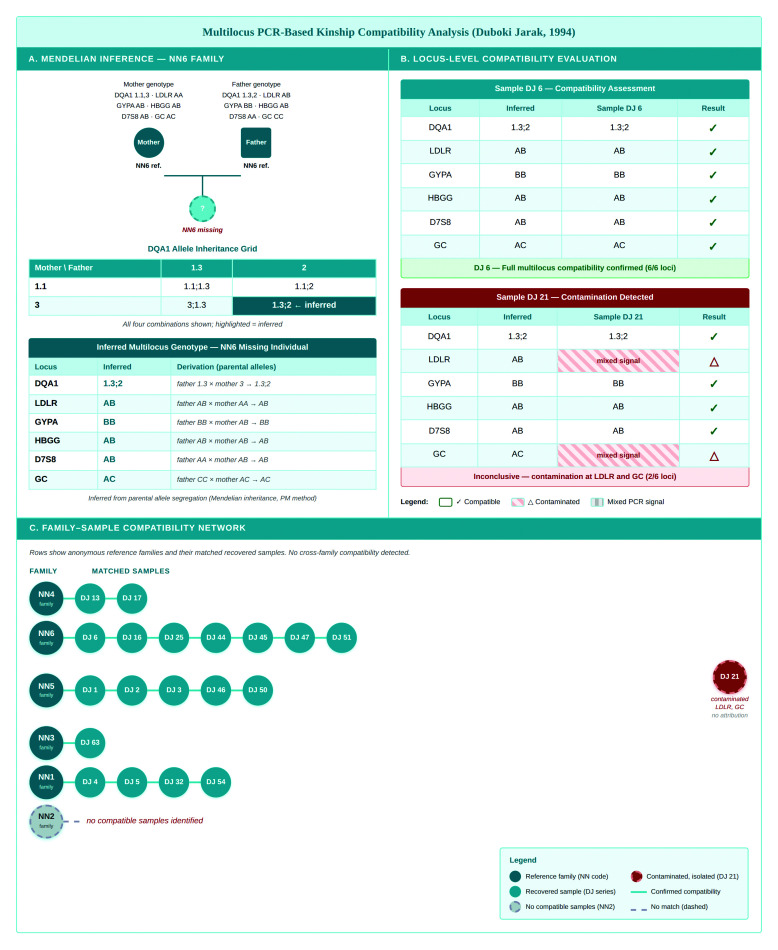
Multilocus polymerase chain reaction (PCR)-based kinship compatibility analysis between reference families and recovered samples from Duboki Jarak. (**A**) Mendelian inference of the multilocus genotype of a missing individual using parental reference profiles (example: NN6 family). Possible offspring genotypes were derived from parental allele segregation at the DQA1 locus (illustrative inheritance grid shown), followed by multilocus genotype construction across DQA1 and Polymarker loci (LDLR, GYPA, HBGG, D7S8, GC). (**B**) Locus-by-locus comparison between inferred family genotype and recovered sample profiles. Full multilocus compatibility (all six loci concordant) is indicated by green check marks. Mixed PCR signals consistent with contamination (DJ 21, LDLR, and GC loci) are highlighted in red, resulting in inconclusive attribution. (**C**) Bipartite network illustrating multilocus compatibility between reference families and recovered samples. Nodes on the left represent families of missing persons; nodes on the right represent recovered samples (DJ numbers). Edges indicate complete concordance across all six loci. No cross-family compatibility was observed. The NN2 family showed no matching samples. Sample DJ 21 is displayed separately due to contamination affecting two loci. Abbreviations: DQA1 – HLA-DQA1 locus; LDLR – low-density lipoprotein receptor locus; GYPA – glycophorin A locus; HBGG – hemoglobin gamma locus; D7S8 – chromosome 7 locus; GC – group-specific component locus.

Following identification of five distinct multilocus genotypes among the successfully analyzed samples, reference profiles obtained from relatives were used to evaluate kinship compatibility. For each family, the possible genotype of the missing individual was inferred from observed parental allele combinations according to Mendelian segregation principles (Figure 4, Panel A). This allowed the generation of a complete multilocus genotype for comparison with the recovered profiles.

Locus-by-locus comparison demonstrated unambiguous multilocus concordance between inferred family genotypes and specific recovered sample clusters (Figure 4, Panel B). The genotype designated as profile 2 (DQA1 1.3;2; LDLR AB; GYPA BB; HBGG AB; D7S8 AB; GC AC) was fully compatible with the NN6 family. All loci were concordant without exclusions, and seven samples (DJ 6, DJ 16, DJ 25, DJ 44, DJ 45, DJ 47, and DJ 51) were attributed to this family cluster.

Profile 1 (DQA1 1.2;4; LDLR AB; GYPA AB; HBGG AB; D7S8 AB; GC AC) corresponded to the NN4 family and was observed in samples DJ 13 and DJ 17. Profile 3 was consistent with the NN5 family and was detected in DJ 1, DJ 2, DJ 3, DJ 46, and DJ 50. Profile 4 matched the NN1 family and was identified in DJ 4, DJ 5, DJ 32, and DJ 54. Profile 5 corresponded to the NN3 family and was present in sample DJ 53.

Importantly, no multilocus genotype demonstrated compatibility with more than one family. The bipartite compatibility network (Figure 4, Panel C) illustrates this clustering structure, showing discrete family-sample groupings without cross-family overlap. This absence of inter-family compatibility supports the internal consistency of genotype inference and strengthens attribution of recovered remains to individual family units.

For the NN2 family, no recovered genotype fulfilled multilocus compatibility criteria. Consequently, no sample could be attributed to this family based on the available genetic evidence.

One specimen (DJ 21) exhibited genotype concordance at the DQA1 locus; however, repeated amplification demonstrated mixed PCR signals at the LDLR and GC loci, consistent with contamination. Because two of six loci were affected, reliable multilocus attribution was not possible (Figure 4, Panel B), and the sample was excluded from definitive kinship assignment.

Overall, integration of multilocus PCR profiles with family reference genotypes allowed resolution of five genetically distinct individuals and enabled consistent family-level attribution of compatible remains. The final DNA results were obtained less than two months after the material was received (end of May to mid-July).

## Discussion

This archival reconstruction documents one of the earliest applications of PCR-based DNA profiling in the identification of victims of a mass-fatality event under active wartime conditions, completed within months of the incident in 1994. The case demonstrates that Croatia implemented molecular identification methods operationally as a DVI tool at a time when DNA was still emerging internationally and before DNA analysis was formally integrated into international DVI protocols (4,12).

The findings must be interpreted within the institutional context of the Croatian Homeland War (27,28), when the country faced continuous humanitarian pressure to identify missing persons while simultaneously building new forensic and health care capacities. Unlike peacetime mass-fatality incidents managed in stable systems, identification procedures in Croatia were conducted amid damaged infrastructure, limited resources, and urgent operational demands (26). In this setting, the rapid implementation of DNA-supported identification was not a gradual technological upgrade, but a necessity-driven innovation aimed at resolving complex, highly fragmented, and thermally altered remains typical of warfare and explosions.

In 1994, PCR-based forensic DNA technology was acquired (including the GeneAmp PCR System 9600 thermal cycler manufactured by Perkin Elmer), which enabled the operational introduction of PCR-based DNA typing in Croatia. This system was installed in a newly established DNA laboratory at the University Hospital of Split, where it was used to support the identification of victims of the war (23-26,30-32). Equally indispensable to the identification process was the Institute of Forensic Medicine in Zagreb, whose experts participated in the initial scene examination and received one victim for autopsy, as were the military judicial authorities, whose formal oversight provided the legal framework within which all forensic and laboratory procedures were conducted. These early PCR marker panels, although limited compared with modern short tandem repeat (STR) kits, were specifically suited for degraded biological material and represented a practical bridge between classical serological methods and high-resolution STR-based identification. In this case, repeated purification and amplification attempts in response to severe degradation reflect both the technical constraints of the period and the methodological maturity required to obtain interpretable genotypes from blast- and heat-damaged tissues.

The technical and procedural outcomes registered in the archived records underline the dual role of early DNA typing: first, as a tool for individualization through multilocus genotype clustering of body parts; and second, as a structured kinship-based confirmation method using reference samples from relatives. The observed separation of multilocus profiles into discrete genotype groups, without cross-family compatibility, supports the internal coherence of the archival identification conclusions. At the same time, the documentation also illustrates typical limitations of early PCR-era forensic genetics, including failure of amplification in a substantial proportion of highly carbonized specimens and at least one instance of mixed signals consistent with contamination. These constraints are expected in early-generation systems and in samples affected by high temperature, decomposition, and blast disruption.

This study suffers from limitations inherent to retrospective archival research. The reconstruction relied exclusively on preserved written and graphic records, without access to raw electrophoretic data or the possibility of re-analysis using modern STR or mtDNA methods. Consequently, the present work did not re-test identifications but evaluated the procedural and technological significance of an early DNA-supported identification workflow within its historical context. Despite these limitations, the consistency of sample labeling, chronological documentation, and genotype-family concordance patterns confirms that molecular genetics was successfully operationalized as part of Croatia’s medico-legal response to mass fatalities in 1994 (23-26,28,32,33).

Taken together, the case supports the conclusion that Croatia introduced and applied forensic DNA technology for mass-fatality identification remarkably early, under conditions of war, and in a manner that anticipated later international DVI standardization (31). Beyond its local significance, this reconstruction contributes to the historiography of forensic genetics by documenting how molecular identification moved from pioneering criminal casework into operational mass-fatality practice – not only in stable Western systems (34,35) but also in conflict-affected settings where the humanitarian imperative to identify the dead accelerated technological adoption.
